# Long-Read Sequencing Annotation of the Transcriptome in DNA-PK Inactivated Cells

**DOI:** 10.3389/fonc.2022.941638

**Published:** 2022-08-02

**Authors:** Liwei Song, Mengjun Yu, Renjing Jin, Meng Gu, Ziyu Wang, Dailun Hou, Shaofa Xu, Jinghui Wang, Teng Ma

**Affiliations:** ^1^ Cancer Research Center, Beijing Chest Hospital, Capital Medical University, Beijing Tuberculosis and Thoracic Tumor Research Institute, Beijing, China; ^2^ Department of Thoracic Surgery, Beijing Chest Hospital, Capital Medical University, Beijing Tuberculosis and Thoracic Tumor Research Institute, Beijing, China; ^3^ Department of Radiology, Beijing Chest Hospital, Capital Medical University, Beijing Tuberculosis and Thoracic Tumor Research Institute, Beijing, China; ^4^ Department of Medical Oncology, Beijing Chest Hospital, Capital Medical University, Beijing Tuberculosis and Thoracic Tumor Research Institute, Beijing, China

**Keywords:** long-read sequencing, DNA-PK, transcriptome, short-read sequencing, alternative splicing

## Abstract

The DNA-dependent protein kinase catalytic subunit (DNA-PKcs) with a Ku70/Ku80 heterodimer constitutes the intact DNA-PK kinase, which is an upstream component of the DNA repair machinery that signals the DNA damage, orchestrates the DNA repair, and serves to maintain genome integrity. Beyond its role in DNA damage repair, the DNA-PK kinase is also implicated in transcriptional regulation and RNA metabolism, with an illuminated impact on tumor progression and therapeutic responses. However, the efforts to identify DNA-PK regulated transcriptomes are limited by short-read sequencing to resolve the full complexity of the transcriptome. Therefore, we leveraged the PacBio Single Molecule, Real-Time (SMRT) Sequencing platform to study the transcriptome after DNA-PK inactivation to further underscore the importance of its role in diseases. Our analysis revealed additional novel transcriptome and complex gene structures in the DNA-PK inactivated cells, identifying 8,355 high-confidence new isoforms from 3,197 annotated genes and 523 novel genes. Among them, 380 lncRNAs were identified. We validated these findings using computational approaches and confirmatory transcript quantification with short-read sequencing. Several novel isoforms representing distinct splicing events have been validated through PCR experiments. Our analyses provide novel insights into DNA-PK function in transcriptome regulation and RNA metabolism.

## Introduction

DNA-PK is the critical component of the cellular response to DNA damage and an essential player in maintaining genome integrity. It is a serine/threonine protein kinase complex composed of the Ku heterodimer proteins (Ku70/Ku80) and the catalytic subunit DNA-PKcs, which belongs to the phosphatidylinositol 3-kinase related kinase (PIKK) family together with Ataxia–telangiectasia mutated (ATM) and RAD3 related (ATR). DNA-PKcs plays multi-faceted roles in non-homologous end joining (NHEJ), DSB repair pathway of choice, DNA replication stress and cell cycle checkpoints (1–3). Besides, increasing evidence has shown the interplay between DNA-PK/DNA-PKcs and noncoding RNAs in DNA damage response (4–7).

Indeed, DNA-PK was identified as part of SP1 transcription complexes (8) and as a regulatory component of the RNA polymerase II complex three decades ago (9). The interaction between transcription and the DNA damage repair machinery involving DNA-PK has been extensively studied (10–13). Recently, several studies have revealed the critical functions of DNA-PK in tumor progression and therapeutic response beyond the DNA repair process, referring to its role in transcription regulation (14–18). DNA-PKcs is critical for autoimmune regulator (AIRE) mediated transcription of toll-like receptors, which play functions in the innate immune system. DNA-PKcs is also required for p53-dependent transcription and for expression of hormone receptors, including androgen receptor and estrogen receptor. Identification of the roles of DNA-PK in cancer-related processes prompted targeting of DNA-PK as a therapeutic advance.

Although the relationship between DNA-PK and the RNA polymerase II complex has been well established, the complex transcriptome dependent on DNA-PK is unknown and much remains to be deciphered about the cellular consequences of DNA-PK. In the past, short-read sequencing-based technology may not have fully resolved the transcriptome landscape dependent on DNA-PK such as alternative splicing (19), alternative transcription initiation, or alternative transcription termination sites and may further hamper the understanding of DNA-PK functions. Long-read sequencing can directly obtain the full-length transcripts, define novel transcribed regions, and discriminate highly similar isoforms of annotated genes.

In this study, we employed the highly accurate PacBio SMRT Sequencing platform to resolve the transcriptome dependent on DNA-PK activity. Our results provide a novel and complex view of the cellular transcriptome centered on a critical transcription regulator. Our study will also expand the understanding of DNA-PK function in both DNA damage repair and transcription.

## Methods

### Cell Culture and Treatment

NCI-H1688 cells were cultured in RPMI-1640 medium supplemented with 10% FBS at 37°C with 5% CO_2_, and were authenticated by karyotype analysis or short tandem repeat analysis and were verified to be free of mycoplasma contamination by PCR. Cells were treated with 1 μM NU7441 (cat#S2638, Selleck, China) for 24 h and subjected to RNA isolation.

### Library Preparation and Sequencing

Total RNAs from DMSO and NU7441-treated NCI-H1688 cells were collected for PacBio SMRT library generation. RNA quality was assessed by an Agilent Bioanalyzer, and samples with RIN scores of >9 were retained. The Iso-Seq library was prepared according to the Isoform Sequencing protocol (Iso-Seq) using the Takara SMARTer PCR cDNA Synthesis Kit (cat #634925 or 634926, Takara, Japan) and the BluePippin Size Selection System protocol as described by Pacific Biosciences (PN 100-092-800-03).

## Data analysis

### Data Processing

Sequence data were processed using the SMRTlink 5.0 software. Circular consensus sequence (CCS) was generated from subread BAM files, parameters: min_length 200, max_drop_fraction 0.8, no_polish TRUE, min_zscore-9999, min_passes 1, min_predicted_accuracy 0.8, max_length 18,000. CCS. BAM files were output, which were then classified into full length and non-full length reads using the pbclassify.py script, ignore polyA false, and minSeq Length 200. Non-full length and full-length fasta files produced were then fed into the cluster step, which involves isoform-level clustering (ICE), followed by final Arrow polishing, hq_quiver_min_accuracy 0.99, bin_by_primer false, bin_size_kb 1, qv_trim_5p 100, and qv_trim_3p 30. Error correction using Illumina reads. Additional nucleotide errors in consensus reads were corrected using the Illumina RNA-seq data with the software LoRDEC.

### Mapping to the Reference Genome

Aligning consensus reads to reference using GMAP with parameters –no-chimeras – cross-species –expand-offsets 1-B 5-K 50000-f samse-n 1 against reference genome.

### Gene Structure Analysis

Gene structure analysis was performed using the TAPIS pipeline. The GMAP output bam format file and gff/gtf format genome annotation file were used for gene and transcript determination. Then, alternative splicing events and alternative polyadenylation events were then analyzed. Fusion transcripts were determined as transcripts mapping to two or more long-distance range genes and were validated by at least two Illumina reads. Unmapped transcripts and novel gene transcript functional annotation. Unmapped transcripts and novel gene transcript functions were annotated based on the following databases: NR (NCBI non-redundant protein sequences); NT (NCBI non-redundant nucleotide sequences); Pfam (Protein family); KOG/COG (Clusters of Orthologous Groups of proteins); Swiss-Prot (A manually annotated and reviewed protein sequence database); KO (KEGG Ortholog database); and GO (Gene Ontology).

We used the software BLAST and set the e-value ‘1e−10’ in NT database analysis. We used the software Diamond BLASTX and set the e-value ‘1e−10’ in NR KOG Swiss-Prot KEGG database analysis. We used the software Hmmscan in our Pfam database analysis.

### Quantification of Transcript Expression

Cuffdiff (v2.1.1) was used to calculate FPKMs of all transcripts in each sample. Isoform FPKMs were computed by summing the FPKMs of transcripts in each gene group. FPKM means fragments per kilo-base of exon per million fragments mapped, calculated based on the length of the fragments and read count mapped to this fragment.

### Differential Alternative Splice

SUPPA was used to calculate the expression weight (Psi) of the alternative splice based on transcript TPM values. A differential alternative splice of the two conditions was performed using the significance test of Psi. The dpsi value was adjusted using the Mann–Whitney U test method. The absolute dpsi value of 0.1 and a p-value of 0.05 were set as the thresholds for significantly differential alternative splices.

### PCR

RNAs from the indicated cell lines were extracted according to the standard procedure. Then, cDNAs were synthesized with 5×PrimeScript RT Master Mix (Perfect Real Time) (Cat. # RR036A, Takara Bio Inc., Japan). After 25–40 cycles of amplification with indicated primers, gel analyses of the PCR product in **Figure 3C** were performed on 1% agarose gels. Primer sequences used are included as part of the **Supplemental Materials**.

## Results

### Experiment Procedure and Long-Read Data Characteristics

**Figure 1:**
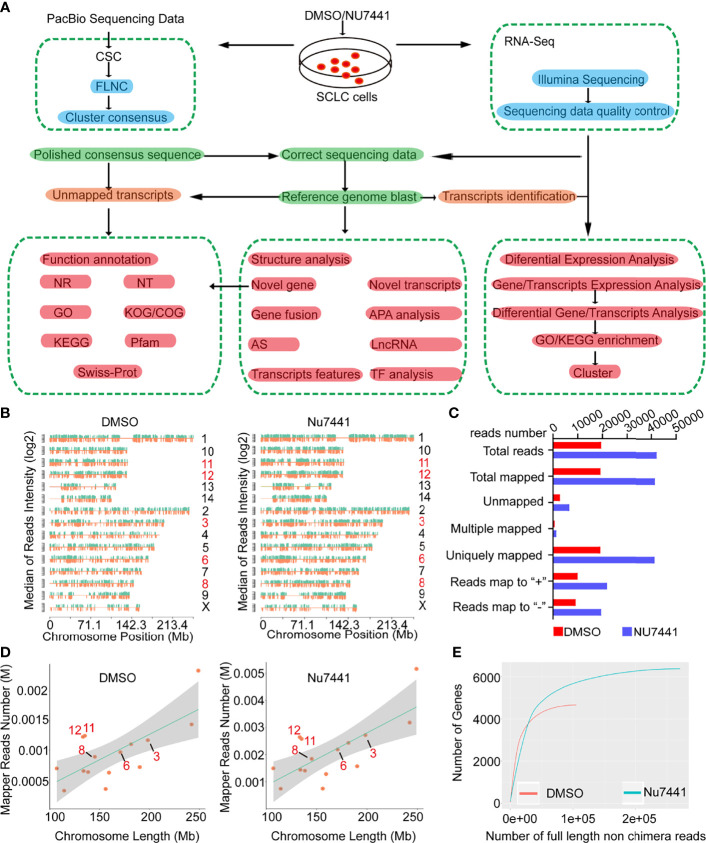
The long-read sequencing flow chart and long-read data characteristics. **(A)** The experimental design and the data processing pipeline of the long-read sequencing. **(B, D)** The chromosomal distribution of sequencing reads between control cells and NU7441 treated cells. **(C)** Summary of the reads mapped to the reference genome. **(E)** The number of genes covered by the long-read sequencing data between control cells and NU7441-treated cells.

To characterize the DNA-PK regulated transcriptome, we followed the experimental and computational pipeline illustrated in **Figure 1A**. The NCI-H1688 cells were treated with 1 μM DNA-PK-specific inhibitor NU7441, and the cells were harvested for RNA extraction. Then, the cDNA libraries were sequenced on the Pacific Biosciences (PacBio) SMRT platform. We also generated a short-read RNA-seq data set collected from the same RNA samples.

Next, the Iso-seq pipeline was used to process the raw downstream data with the official PacBio software package SMRTlink (**Figure 1A** and *Methods*). Then, the polished third-generation reads were corrected by high-quality second-generation data with LoRDEC software (20). To make a preliminary assessment of the DNA-PK dependent long-read transcriptome, we mapped the dataset to the reference genome using GMAP (Genomic Mapping and Alignment Program). The density of total mapped transcripts mapped to individual chromosomes (by positive and negative strands) on the genome was counted (**Figure 1B**). 19469 and 42125 transcripts were successfully mapped to the reference genome for the DMSO and NU7441 treated groups, respectively. Only around 1.5% of the dataset was unmapped (**Figure 1C**), indicating the high quality of the dataset. We also counted different depths of full-length transcripts and the number of genes in their maps for saturation curve analysis. The results indicated that the amount of sequencing data is sufficient for subsequent analysis (**Figure 1D, E**).

### Functional Characterization of the Long-Read Data

**Figure 2:**
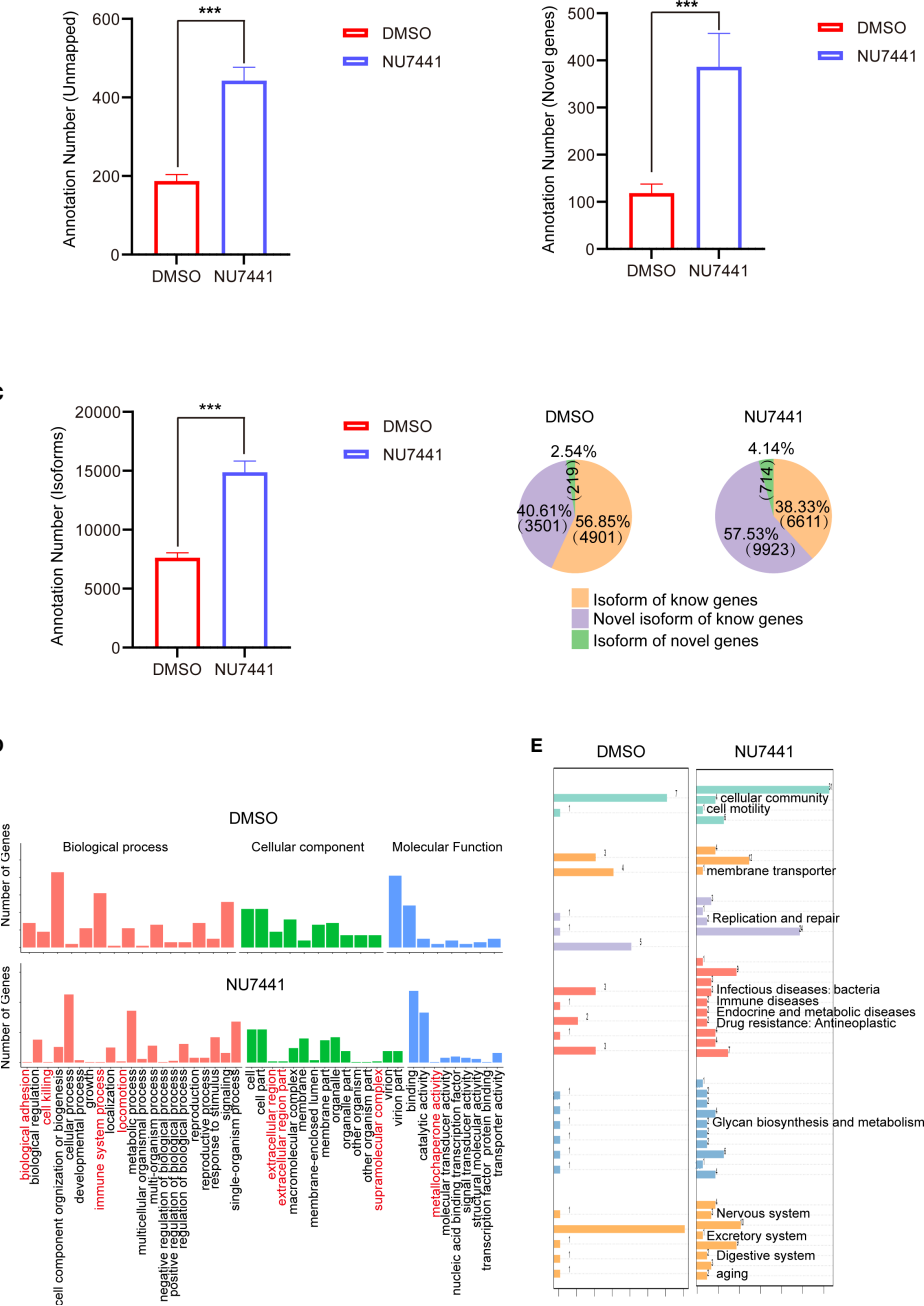
Annotations of the long-read sequencing data. **(A)** Annotations of the unmapped transcripts in control cells and NU7441-treated cells (*p <*0.001). **(B)** Annotations of the novel genes in control cells and NU7441-treated cells (*p <*0.001). **(C)** Annotations of the isoforms in control cells and NU7441-treated cells in (*p <*0.001). **(D)** GO analysis of novel genes identified in control cells and NU7441-treated cells. **(E)** KEGG analysis of novel genes identified in control cells and NU7441-treated cells.

To obtain comprehensive annotation information, the transcripts unmapped to the reference genome, novel genes and the transcript isoforms were annotated by seven databases (NR, Non-Redundant Protein Database; NT, NCBI GenBank, EMBL and DDBJ databases; Pfam; KOG/cog, Cluster of Orthologous Groups of proteins; Swissprot; KEGG, Kyoto Encyclopedia of Genes and Genomes; and GO, Gene Ontology). The numbers of transcripts successfully annotated for the DMSO and NU7441 groups are summarized in **Figures 2A–C**. The results indicated that NU7441 treatment elicited significant transcriptome changes.

We are particularly interested in the functional annotations of novel genes after NU7441 treatment. According to the mapping results between the transcript and the reference genome, the reads compared to the unannotated region of the reference genome GTF file are defined as a novel gene. Based on the GO database, novel genes involved in biological adhesion, cell killing, immune system processes, icomotion, extracellular region, and metallochaperone activity are increased in the NU7441 treated transcriptome (**Figure 2D**). Based on the KEGG annotation, novel genes involved in cellular community, cellular motility, membrane transporter, replication and repair, infectious disease, immune diseases, endocrine and metabolic diseases, drug resistance, glycan biosynthesis and metabolism, nervous system, excretory system, digestive system, and aging are increased in the NU7441 treated transcriptome (**Figure 2E**).

### Transcriptome Structure Analysis and Alternative Splicing Profiling in the Long-Read Data

**Figure 3:**
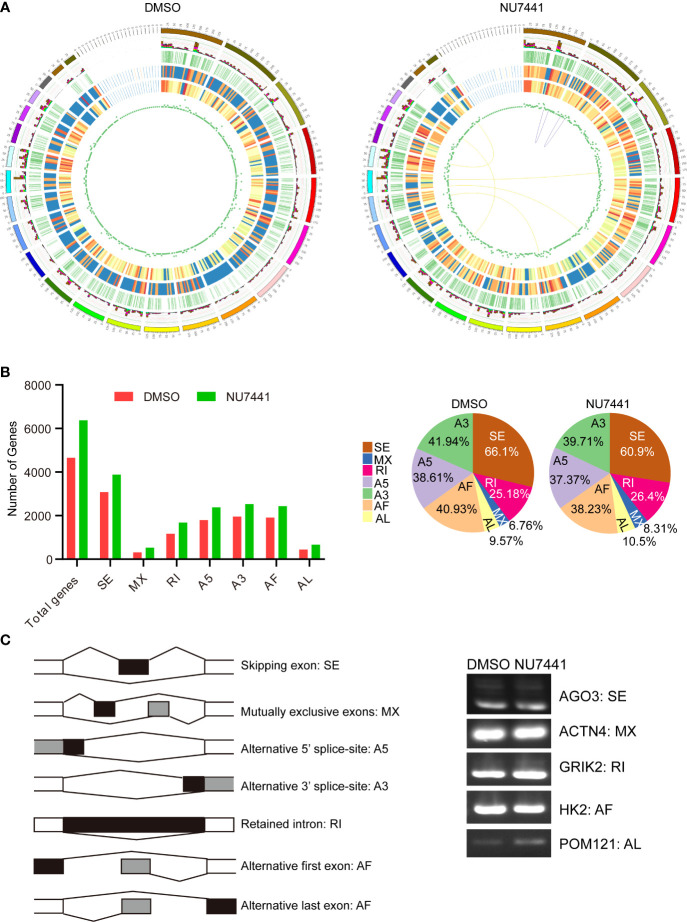
Transcriptome structure analysis and alternative splicing profiling in the long-read data. **(A)** The circus display of chromosome sequence; alternative splicing loci (light blue for intron retention, green for A3 alternative splicing, yellow for A5 alternative splicing, purple for exon skipping, red for mutual exclusive exons, brown for alternative first exon, dark blue for alternative last exon); alternative polyadenylation loci; the distribution of new transcripts; novel gene distribution; lncRNA density distribution; gene fusions, purple line (same), yellow line (different) genes on chromosomes fused. **(B)** Alternative splicing patterns in the long-read data. The different splicing patterns were depicted in the left panel. The number of genes with different splicing events between control cells and NU7441-treated cells were summarized. The percentage of different splicing patterns was also shown in the pie chart. **(C)** Validation of the alternative splicing events in long-read data. Different splicing events for *AGO3*, *ACTN4*, *GRIK2*, *HK2*, and *POM121* were validated by qPCR.

Circos was used to visualize the transcriptome structure before and after DNA-PK inactivation (**Figure 3A**). From outside to inside, different circus represented chromosome sequence; alternative splicing loci (stacked bar chart with different colors for different variable splice types; the NU7741 treated cells showed 60.9% exon skipping compared with 66.1% of control cells); alternative polyadenylation loci; the distribution of new transcripts; the closer to red, the higher the density; novel gene distribution, the closer to red, the higher the density; lncRNA density distribution; gene fusions, purple line (same), yellow line (different) genes on chromosomes fused. Based on the Circos analysis, it is clearly seen that NU7441 treatment induced a significant increase in new transcripts and novel gene distribution. Meanwhile, 7 gene fusions were identified, possibly due to transcription-mediated splicing (21).

Alternative splicing is an important mechanism for regulating gene expression and generating proteomic diversity (22). In a previous study, we have shown that the inactivation of DNA-PK activity regulates CD44 alternative splicing (19). In the long-read data, we attempted to gain an alternative splicing landscape in DNA-PK inactivation cells. The transcriptome data were processed using the SUPPA software (23). The results showed that more genes had alternative splicing events in the NU7441 treated cells with an increasing trend in AL (alternative last exon), MX (mutually exclusive exon), and IR (intron retention), the latter emerging as a promising cancer treatment target (**Figure 3B**) (24).

Next, we used exon-specific PCR to validate several splicing events detected in the long-read data. The PCR primers are shown schematically (**Figure 3C**; see **Supplemental Table S1**). The following events detected in long-read data were confirmed by PCR: skipped exon (*AGO3*), mutually exclusive exon (*ACTN4*), retained intron (*GRIK2*), the alternative first exon (*HK2*), and alternative last exon (*POM121*). As detected in the long-read data for these examples, the splice site usage seen by PCR differs in untreated and DNA-PK inactivated cells. Therefore, our approach to identifying novel isoforms of transcripts in DNA-PK inactivated cells replenishes the current understanding of DNA-PK function.

### Differential Gene Analysis in Short-Read Data

**Figure 4:**
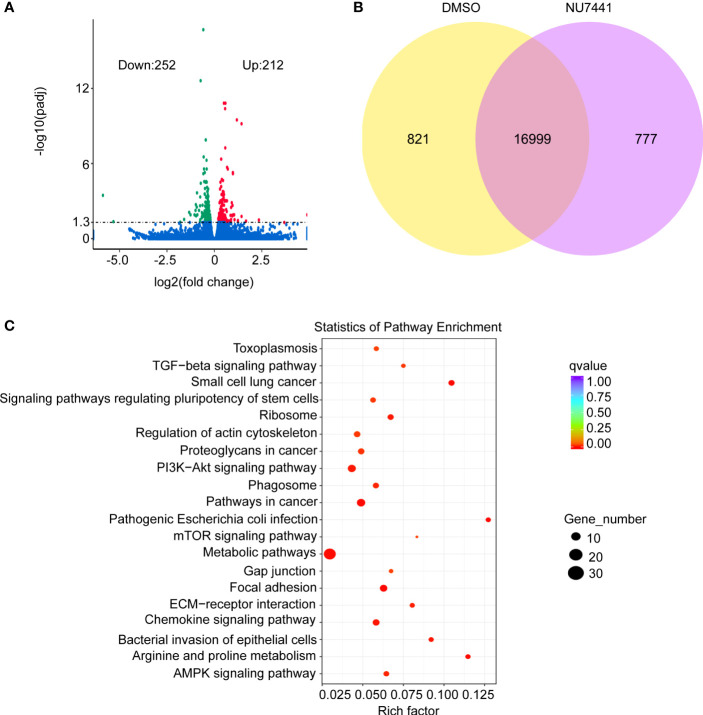
Differential expression of genes analysis in the short-read data. **(A)** Volcano plot for differentially expressed genes between control and NU7441-treated cells. **(B)** Venn diagram for differentially or co-expressed genes between control and NU7441-treated cells. **(C)** Functional enrichment of the differentially expressed genes between control and NU7441-treated cells.

To gain an insight of the differential genes based on expression level between control cells and DNA-PK inhibited cells, the short-read data were analyzed. As shown in the volcano plot, 212 genes were upregulated and 252 genes were downregulated after NU7441 treatment (**Figure 4A**). Differentially and co-expressed genes were shown as Venn Diagrams (**Figure 4B**). A total of 16,999 genes were shared between the control cells and NU7441 treated cells. A total of 821 genes were expressed and 777 genes were differentially expressed between control cells and the NU7441-treated cells. Pathway enrichment analysis indicated that chemokine signaling pathways and metabolic pathways were enriched after DNA-PK inhibition (**Figure 4C**).

## Discussion

Here, our findings illustrate that DNA-PK has a critical impact on global transcription in mammalian cells. Through long-read sequencing technology, many more transcripts were discovered, including novel genes, novel isoforms, and novel lncRNAs. Our discoveries will shed light on the understanding of cancer phenotypes associated with DNA-PK, such as tumor progression and unfavorable prognosis in patients with high DNA-PK expression (17, 18, 25). It will also facilitate the depiction of potential underlying mechanisms associated with DNA-PK and finally illuminate the future targeting of DNA-PK in human malignancies.

Based on our findings, DNA-PK kinase activity is essential for the global transcriptome landscape. It is known that DNA-PK is a holoenzyme composed of a heterodimer of Ku70/Ku80 and DNA-PKcs. Posttranslational modifications of DNA-PKcs, including phosphorylation, play important roles in DNA-PK activation or kinase activity (3, 26). How precisely DNA-PKcs is activated, especially during the transcription process, is still mysterious. Ku70/Ku80 and DNA ends in DNA repair provide the prerequisites for DNA-PKcs activation. However, increasing pieces of evidence implicate the Ku-independent process. It would be interesting to elucidate other co-factors that assist DNA-PK or DNA-PKcs activation during the transcription process, such as transcription-coupled DNA repair machinery. It is also feasible to identify DNA-PKcs phosphorylation-dependent interaction factors in transcription. The temporospatial distribution of DNA-PK or DNA-PKcs containing a transcription body should elaborate on the function of DNA-PK.

Furthermore, cancer context-dependent mechanisms of DNA-PK activity are also worthy of investigation. Specific co-factors involved in DNA-PKcs–mediated transcriptional regulation in cancer cells and the specific local chromatin structure required for DNA-PKcs-dependent transcription need to be elucidated. Though our long-read sequencing data identified numerous novel transcripts, how DNA-PKcs dictates the selection of the transcriptome is unknown. Further technologies such as ChIP-Seq and 3C chromatin capture are needed to investigate the details of the chromatin context of DNA-PK regulated transcription. Why can DNA-PK inhibition cause such a large number of transcriptions? DNA-PK activity may exert a suppressive role on RNA Pol II, or the DNA-PK function in DNA replication repair may suppress the transcription. It needs further validation. Alternatively, the roles of DNA-PK in DNA repair and transcription may be different. Therefore, separate targeting of the different roles of DNA-PK by inhibitors may provide treatment tactics for cancer. Furthermore, although the DNA-PK inhibitors inhibit the kinase activity of the protein, little is known about kinase-independent functions of DNA-PKcs that may affect therapeutic efficacy in targeting malignancy.

The functional consequences of such a novel transcriptome induced by DNA-PK inhibition are also interesting projects to work on. Though preliminary GO and KEGG analysis have been performed, the novel genes and novel lncRNAs in the DNA-PK network and critical for DNA-PK kinase activity and function in cells are to be elucidated. Furthermore, the DNA-PK mutations identified in many cancer types should also be considered in the context of transcriptional regulation and cancer progression. It will be critical to confirm whether modulators and/or substrates of DNA-PKcs change as cancers progress and mutate. It will affect the design of specific therapeutic strategies targeting specific stages of disease.

In summary, DNA-PK plays a pleiotropic role impacting human malignant phenotypes. Each new advance may define a novel therapeutic strategy for any number of cancer types.

## Data Availability Statement

The datasets presented in this study can be found in online repositories. The names of the repository/repositories and accession number(s) can be found below: https://www.ncbi.nlm.nih.gov/bioproject/849578.

## Ethics Statement

Ethical review and approval was not required for the study on human participants in accordance with the local legislation and institutional requirements. Written informed consent from the participants was not required to participate in this study in accordance with the national legislation and the institutional requirements.

## Author Contributions

Concept and design: TM, SX, and JW. Administrative support: MG and ZW. Collection and assembly of data: LS, MY, and RJ. Data analysis and interpretation: DH, SX, JW, and TM. Manuscript writing: all authors. Final approval of manuscript: all authors.

## Conflict of Interest

The authors declare that the research was conducted in the absence of any commercial or financial relationships that could be construed as a potential conflict of interest.

## Publisher’s Note

All claims expressed in this article are solely those of the authors and do not necessarily represent those of their affiliated organizations, or those of the publisher, the editors and the reviewers. Any product that may be evaluated in this article, or claim that may be made by its manufacturer, is not guaranteed or endorsed by the publisher.
