# Supplementary material for: Long-Read Sequencing Annotation of the Transcriptome in DNA-PK Inactivated Cells
Source: Front Oncol. 2022 Aug 2;12:941638. doi: 10.3389/fonc.2022.941638 (PMC9382581; doi:10.3389/fonc.2022.941638)
Supplement: Supplementary file 1 [file DataSheet_1.zip › Source Data/Original data for Figure 3C.pptx]

## Slide 1
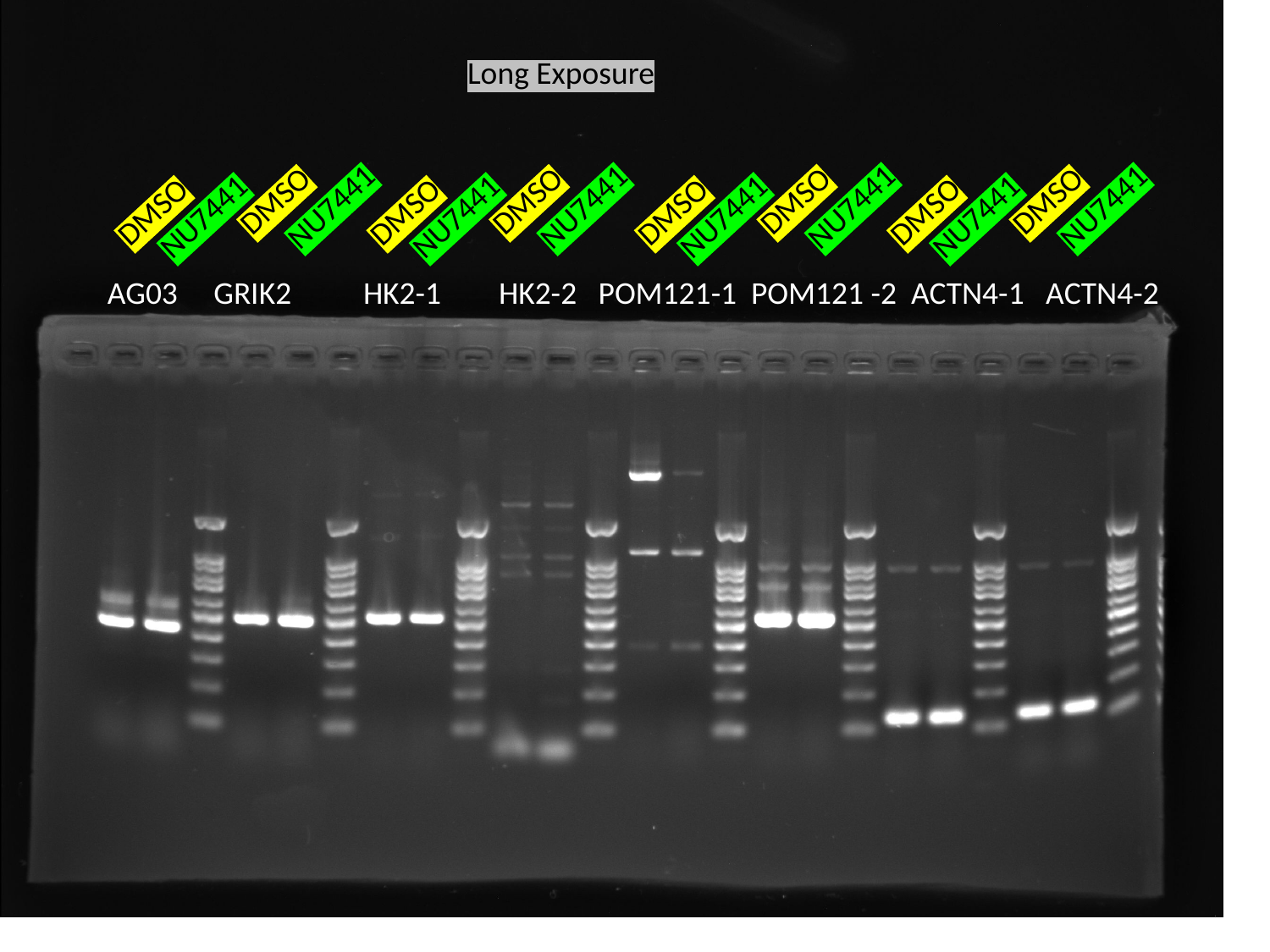

Long Exposure
DMSO
DMSO
DMSO
DMSO
NU7441
NU7441
NU7441
NU7441
DMSO
DMSO
DMSO
DMSO
NU7441
NU7441
NU7441
NU7441
AG03 GRIK2 HK2-1 HK2-2 POM121-1 POM121 -2 ACTN4-1 ACTN4-2

## Slide 2
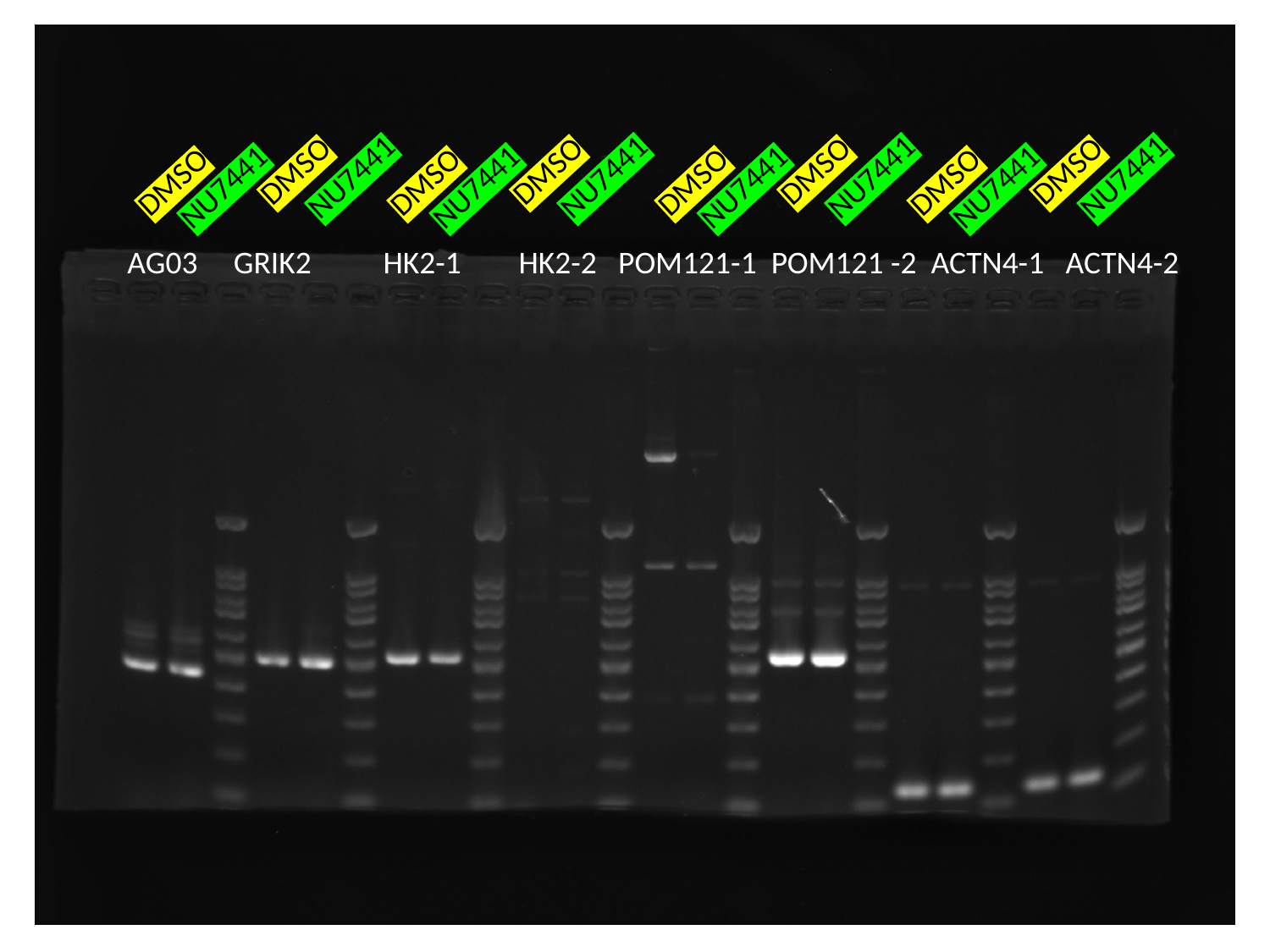

DMSO
DMSO
DMSO
DMSO
NU7441
NU7441
NU7441
NU7441
DMSO
DMSO
DMSO
DMSO
NU7441
NU7441
NU7441
NU7441
AG03 GRIK2 HK2-1 HK2-2 POM121-1 POM121 -2 ACTN4-1 ACTN4-2
