# Supplementary material for: Long-Read Sequencing Annotation of the Transcriptome in DNA-PK Inactivated Cells
Source: Front Oncol. 2022 Aug 2;12:941638. doi: 10.3389/fonc.2022.941638 (PMC9382581; doi:10.3389/fonc.2022.941638)
Supplement: Supplementary file 4 [file Presentation_1.pdf]

Supplementary Method:

Statistical analysis

The results are expressed as mean±standard error of mean. The significance of the differences between two groups were used Student's t-test. The p-values ≤ 0.05 were considered significant.
